# A Novel N/P-Doped Carbon Shells/Mn_5.64_P_3_ with Hexagonal Crystal Structure Hybrid as a Prospective Anode for Lithium-Ion Batteries

**DOI:** 10.3390/molecules30061346

**Published:** 2025-03-17

**Authors:** Fei Wang, Jingxia Gao, Hui Li, Junle Zhang, Aiyun Jiang, Yong Liu, Fengzhang Ren

**Affiliations:** 1 Faculty of Engineering, Huanghe Science & Technology University, Zhengzhou 450006, China; gjx0501@hhstu.edu.cn (J.G.); leehui@hhstu.edu.cn (H.L.); zhangjunle1999@163.com (J.Z.); jiangaiy@hhstu.edu.cn (A.J.); 2 School of Materials Science and Engineering, Henan University of Science and Technology, Provincial and Ministerial Co-Construction of Collaborative Innovation Center for Non-Ferrous Metal New Materials and Advanced Processing Technology, Luoyang 471023, China; renfz@haust.edu.cn; 3 Henan Key Laboratory of Non-Ferrous Materials Science & Processing Technology, Luoyang 471023, China

**Keywords:** Mn_5.64_P_3_, carbon shells, hexagonal crystal structure, lithium-ion batteries, phase structure

## Abstract

The tailored crystalline configuration coupled with submicron particles would be conducive to superior ionic conductivity, which could further improve the cycle life of lithium-ion batteries (LIBs). Here, manganese phosphide (Mn_5.64_P_3_) particles with hexagonal crystal structure embedded into nitrogen/phosphorus (N/P) co-doped carbon shells (Mn_5.64_P_3_-C) are successfully prepared by the self-template and recrystallization–self-assembly method. The electrochemical properties of as-synthesized Mn_5.64_P_3_-C as anode materials for LIBs are systematically investigated. The XRD and HRTEM combined with SAED indicate that the prepared Mn_5.64_P_3_-C hybrid with the ratio of 1:10 of Mn:C present a hexagonal crystal structure covered with a carbon layer. During charging/discharging at the current density of 0.5 A g^−1^, the Mn_5.64_P_3_-C electrode exhibits the reversible capacity of 160 mAh g^−1^ after 3000 cycles with high-capacity retention. The ex-situ XRD of initial discharge/charge process at different voltages implies that the Mn_5.64_P_3_ could be transformed to the amorphous Li*_x_*Mn*_y_*P*_z_*. The N/P co-doped carbon shells can provide high specific area for electrolyte infiltration, and act as the buffer matrix to suppress the loss of the Mn_5.64_P_3_ active material during cycling. The Mn_5.64_P_3_ with the hexagonal crystal structure and N/P co-doped carbon shells could promote the further optimization and development of manganese phosphide for high-performance LIBs.

## 1. Introduction

Rechargeable lithium-ion batteries (LIBs) are of significance for next generation energy storage systems due to their high energy and power density, fast charge-discharge capability, and long service lifetime [1,2,3]. However, there are still some issues to be addressed in order to meet the performance requirements of LIBs applied in electric vehicles, portable electronics, and smart grids. Those are leading to the increasing demands for high energy density and long cycling life anode materials for the next generation of LIBs, such as oxides [4,5], alloys and compounds [6,7,8], nitrides [9,10], sulphides [11,12] and phosphides [13,14]. Among them, transition metal phosphides are the most attractive candidates for LIBs due to their higher specific capacity, improved cycling behavior and lower cost, such as CoP [15], MnP [16], ZnP_2_ [17], Sn_4_P_3_ [18], Ni_2_P [19,20], Cu_3_P [21], MoP [22] and FeP [23]. MnP with an orthorhombic crystal structure (space group *Pnma*) could be alloyed with Li and then form Li*_x_*Mn*_y_*P*_z_*, which could display high initial charge/discharge capacities of about 870/1104 mAh g^−1^, but suffer from a rapid degradation of capacity during cycling [24]. The rapid capacity loss could be attributed to slow reaction kinetics, severe volume expansion, and microstructure change.

To solve these issues, great efforts have been made to relieve the severe volume and improve cycling performance of the electrodes. For example, MnP nanoparticles or nanorods were prepared to improve the reaction kinetics of MnP in LIBs [24,25,26]. In the initial discharging process, MnP phase could be transformed to the amorphous Li*_x_*Mn*_y_*P*_z_* and Mn_2_P phases. The residual Li*_x_*Mn*_y_*P*_z_* and Mn_2_P phases in the electrode could act as the buffer matrix to relieve capacity loss during cycling. In addition, Gillot et al. synthesized Li_5.5_Mn_2.5_P_4_ with a tetragonal structure and Li_7_MnP_4_ with a cubic structure through ball-milling lithium, manganese, and phosphorus powders and subsequently annealing at 800 °C in an argon atmosphere [27]. Compared to Li_7_MnP_4_, the Li_5.5_Mn_2.5_P_4_ electrode could nicely maintain the capacity during cycling, which could be attributed to no drastic decomposition of the Li_5.5_Mn_2.5_P_4_ electrode during cycles. This indicates that MnP with suitable crystal structure is conducive to improving the cyclic performance of MnP. Recently, Hong and co-workers investigated the substitutional solid solution Mn_1−*x*_Fe*_x_*P derived from FeP and MnP by high energy mechanical milling, which could effectively relieve volume changes and inhibit the aggregation of the active particles during cycling [28]. In addition, Hong and co-workers synthesized the other substitutional solid solution Mn_1−*x*_V*_x_*P compounds obtained from MnP and VP via similar high energy mechanical milling [29]. The synergistic effect of the alloying electrochemical reaction and insertion hybrid electrochemical reaction in the prepared Mn_1−*x*_V*_x_*P could effectively relieve the volume expansion and prevent the agglomeration of alloying reaction-type Li-Mn-P crystallite during the cycles. Hence, it can enhance the cyclic performance of the electrode, and ensure rapid transport of electron and ion when MnP is modified by orthorhombic crystal structure.

In hexagonal lattice structure, atoms are closely arranged in two-dimensional space to form a “three-sphere empty” closed layer. When these structures are stacked in three-dimensional space, they form a hexagonal cell. Hexagonal enclosure is more conductive, mainly because the empty shape of the enclosure is triangular, which facilitates free flow of electrons and reduces the possibility of scattering. The Mn_5.64_P_3_ with hexagonal crystal structure (*P*6_2_*m*, a = b = 0.60842 nm and c = 0.34595 nm) is proposed as the high-performance anode of LIBs. However, few works have investigated Mn_5.64_P_3_ in alkali metal ion batteries. In addition, the capacity of manganese phosphide would rapidly decay due to particle agglomeration after the cycles. Therefore, designing suitable Mn_5.64_P_3_-based composites with robust structure and favorable conductivity for high-performance LIBs via a convenient and reliable strategy is still challenging.

In this work, we synthesized Mn_5.64_P_3_ with hexagonal crystal structure embedded into nitrogen doped carbon shells (denoted as Mn_5.64_P_3_-C) through self-synthesized precursors and subsequent annealing in argon atmosphere. The Mn_5.64_P_3_-C precursor could be synthesized in one-step preparation process without additional carbon sources or other phosphorous reducing agents or high-boiling organic solvents [30]. The synergistic effect of Mn_5.64_P_3_ with hexagonal crystal structure and nitrogen doped carbon shells endows the Mn_5.64_P_3_-C hybrid with superior electrochemical performance as anode in LIBs. The systematic studies on the structural relation and electrochemical properties of Mn_5.64_P_3_-C hybrid with different carbon source additives are performed as the electrodes in LIBs. The Mn_5.64_P_3_-C hybrid with optimal carbon source additive amounts electrode could display the excellent cycling performance of 160 mAh g^−1^ after 3000 cycles at 0.5 A g^−1^ and rate capability of 115 mAh g^−1^ at 5.0 A g^−1^.

## 2. Results and Discussion

**Figure 1:**
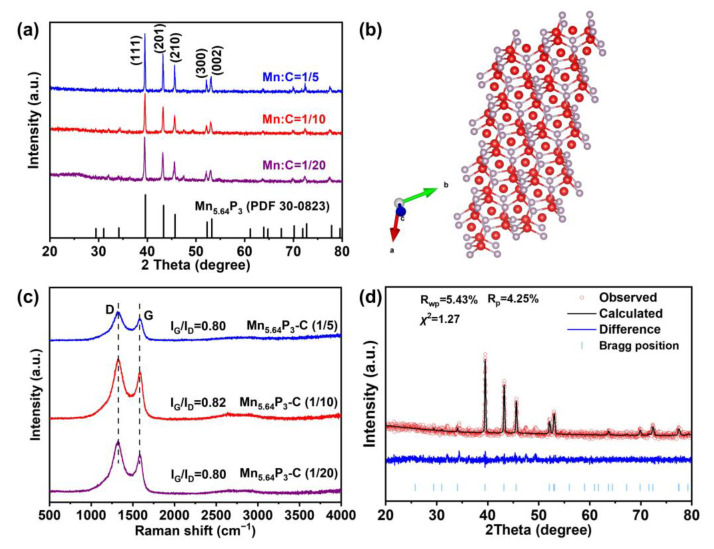
(**a**) XRD patterns of as-synthesized Mn_5.64_P_3_ with various ratio of Mn:C. (**b**) crystal structures of as-synthesized Mn_5.64_P_3_. (**c**) Raman spectra of as-synthesized Mn_5.64_P_3_ with various ratio of Mn: MA. (**d**) XRD Rietveld refinement results of as-synthesized Mn_5.64_P_3_-C (1/10).

Figure 1a shows the XRD patterns of the as-synthesized Mn_5.64_P_3_-C hybrid with different additive amounts of carbon sources (5 mmol, 10 mmol, 20 mmol). It can be seen that the obvious XRD characteristic peaks of all Mn_5.64_P_3_-C hybrid anode materials at 39.62°, 43.34°, 45.72°, 52.28°, and 53.24°, corresponding to the (111), (201), (210), (300), and (002) crystal planes of Mn_5.64_P_3_ (PDF card: 30-0823). It reveals that all Mn_5.64_P_3_-C hybrid anode materials show a hexagonal crystal structure with a space group *P*6_2_*m*. The Mn_5.64_P_3_ and MnP crystal structures are shown in Figure 1b and Figure S1, respectively. Mn_5.64_P_3_ crystallizes in the *P*6_2_*m* space group in which the phosphorus atoms sites in the Wyckoff 3f (1/4, 0, 0) sites forming a hexagonal close-packed (hcp) network. In addition, the 1/3 manganese atoms are in the Wyckoff 2b (0, 0, 1/2) sites forming octahedral coordination environment, and the 2/3 manganese atoms are in the Wyckoff 6i (1/2, 0, 1/4) tetrahedral sites. The octahedral Mn layer and tetrahedral Mn layer are arranged alternately along the C-axis and embedded in the hcp framework constructed by P atoms. The octahedral Mn layer and tetrahedral Mn layer are arranged alternately along the C-axis and embedded in the hcp framework constructed by P atoms. This arrangement gives Mn_5.64_P_3_ unique electron transport properties. In contrast, MnP crystallizes in the space group *Pnma*, in which all the manganese cations are localized in the same site Wyckoff 4c, in which the Mn and P atoms are arranged in the ab plane and are connected by P-P covalent bonds along the C-axis [27].

The carbon structures in Mn_5.64_P_3_-C hybrid with different additive amounts of carbon sources were investigated. Figure 1c presents the Raman spectrum of Mn_5.64_P_3_-C hybrid with different additive amounts of carbon sources. Figure 1c shows the peaks around 1330 and 1580 cm^−1^ corresponding to the disordered structure (D-band) and the ordered graphitic structure (G-band) of the carbon materials, respectively. In addition, the relative intensity ratios of I_D_/I_G_ for these Mn_5.64_P_3_-C hybrid materials are rather high, which imply the high disorder carbon structure in the materials. Among them, the higher value of I_D_/I_G_ in Mn_5.64_P_3_-C hybrid (1/10) indicates higher disorder carbon structure and active sites in Mn_5.64_P_3_-C hybrid (1/10). The Rietveld refinements were conducted on the obtained XRD patterns using GSAS-II version 5768 software (Figure 1d, Figures S2 and S3). The refined lattice constants (a, b, and c) and unit cell volume (V) are shown in Tables S1 and S2. The diffraction patterns of as-synthesized Mn_5.64_P_3_ with various ratio of Mn:C were successfully refined using the Mn_5.64_P_3_ crystal structure (space group *P*-6_2_*m*). From the Rietveld refinement results, the as-synthesized Mn_5.64_P_3_ with various ratio of Mn:C present almost the same atomic coordinates, space occupancy and lattice parameters. The results show obvious structural changes than MnP [28,29], which implying different lithium storage in Mn_5.64_P_3_.

**Figure 2:**
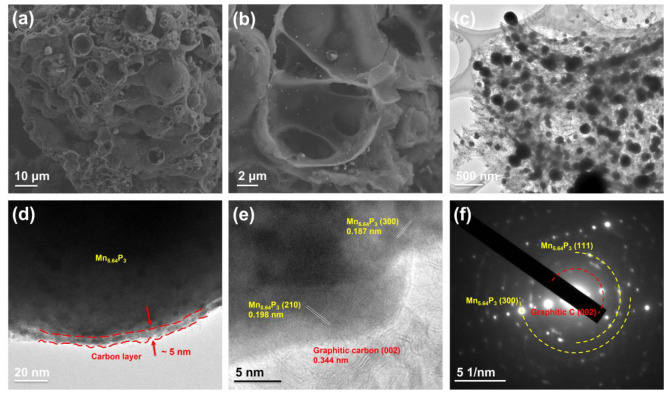
(**a**,**b**) the FESEM images of the as-synthesized Mn_5.64_P_3_ with the ratio of 1:10 of Mn:C. (**c**) TEM image, (**d**,**e**) HRTEM images, (**f**) SAED pattern.

The microstructure of as-synthesized Mn_5.64_P_3_-C hybrid are investigated by FESEM and TEM. As shown in Figure 2a,b, the as-synthesized Mn_5.64_P_3_ hybrid with the ratio of 1:10 of Mn:C (10 mmol carbon source) presents porous microsized shells. In contrast, the as-synthesized Mn_5.64_P_3_ hybrid with the ratio of 1:5 of Mn:C (5 mmol carbon source) in Figure S4 shows the heterogeneous mixture composed of micrometer-sized Mn_5.64_P_3_ particles and carbon materials. In addition, the as-synthesized Mn_5.64_P_3_ hybrid with the ratio of 1:20 of Mn:C (20 mmol carbon source) in Figure S5 shows the broken and irregular pieces. Clearly, Mn_5.64_P_3_ in the as-synthesized Mn_5.64_P_3_ hybrid with the ratio of 1:5 of Mn:C are homogeneously immobilized into porous microsized shells, which is essential to adjust the volume change during cycling. Importantly, such porous microsized shells’ structure can not only produce extra active sites, but also shorten the diffusion pathway of Li ions, thereby favorable for electrochemical reactions.

Furtherly, the distribution of Mn_5.64_P_3_ in the as-synthesized Mn_5.64_P_3_ hybrid with the ratio of 1:10 of Mn:C was investigated. The transmission electron microscopy (TEM) in Figure 2c shows submicron sized Mn_5.64_P_3_ particles are uniformly dispersed in carbon shells, in agreement with the FESEM observations (Figure 2a,b). The high-resolution TEM (HRTEM) image in Figure 2d indicates the submicron sized Mn_5.64_P_3_ particle is further coated with carbon with a thickness of about 5 nm. The HRTEM image (Figure 2e) shows two clear lattice stripes at 0.198 and 0.187 nm, which are consistent with (210) and (300) crystal planes of Mn_5.64_P_3_. In addition, the lattice fringe of the carbon material in Mn_5.64_P_3_ hybrid is 0.344 nm corresponding to the (002) crystal plane of graphite, which implies the high degree of graphitic carbon on the surface of Mn_5.64_P_3_. The selected-area electron diffraction (SAED) pattern in Figure 2f can be indexed to the Mn_5.64_P_3_ phase and graphitic carbon, which display different degrees of crystallization of Mn_5.64_P_3_.

**Figure 3:**
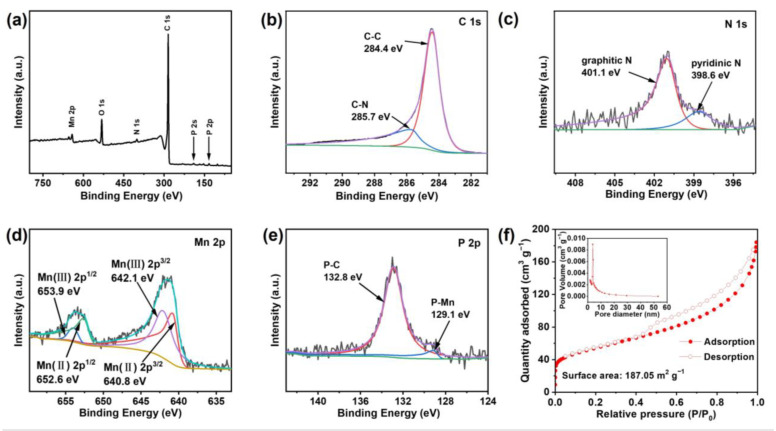
(**a**) XPS spectra of Mn_5.64_P_3_ prepared with Mn:C = 1:10, survey spectrum, (**b**) C 1s spectrum, (**c**) N 1s spectrum, (**d**) Mn 2p spectrum, and (**e**) P 2p spectrum. (**f**) N_2_ adsorption-desorption isotherm and and pore size distributions of Mn_5.64_P_3_ prepared with Mn:C = 1:10.

To reveal the valence state of elements and material composition of the as-synthesized Mn_5.64_P_3_ hybrid with the ratio of 1:10 of Mn:C, X-ray photoelectron spectroscopy (XPS) is conducted. The full survey XPS spectrum (Figure 3a) shows the presence of Mn, P, C, O, and N. The high-resolution C 1s spectrum in Figure 3b can be fitted into two peaks of C-C (284.4 eV), C-N (285.7 eV) [27,28,29]. As shown in Figure 3c, the high-resolution spectrum of the N 1s of the as-synthesized Mn_5.64_P_3_ hybrid could be fitted into two peaks of pyridinic N (398.6 eV) and graphitic N (401.1 eV) [30]. P and N dual doping could synergistically induce a larger number of defects in the as-synthesized Mn_5.64_P_3_ hybrid to enhance the adsorption and storage of Li^+^, thus further improving its electrochemical performance. In Figure 3d, the Mn 2p_3/2_ region displays two peaks at 642.3 and 641.1 eV, which are assigned to Mn^3+^, Mn^2+^, respectively. And the Mn 2p_1/2_ region contains two peaks at 654.1 and 652.7 eV, which are attributed to Mn^3+^, Mn^2+^, respectively. The Mn^3+^, Mn^2+^ in deconvoluted Mn 2p spectrum could be ascribed to nonstoichiometric Mn and P in Mn_5.64_P_3_. Figure 3e shows that the high-resolution P 2p spectrum has three separate peaks at 132.8 and 129.1 eV for P-C and P-Mn [31,32]. It is further confirmed that P is well doped in the carbon layer.

The pore-sized distribution and porous structure of the as-synthesized Mn_5.64_P_3_ hybrid with the ratio of 1:10 of Mn:C were calculated from nitrogen adsorption-desorption isotherms and pore-size distribution curves. Figure 3f exhibits a typical IV isotherms and a large Brunauer-Emmett-Teller surface area (187.05 m^2^ g^−1^), indicating the main existence of mesopore in the as-synthesized Mn_5.64_P_3_ hybrid with high surface area. Pore-size distribution curves (inset in Figure 3f) further confirms the massive mesopores feature centered at a mesopores entrance size of 3.8 nm. In general, the P/N doping and high specific surface areas can provide abundant electrochemically active sites for electrode/electrolyte contact and lithium-ion accommodation.

**Figure 4:**
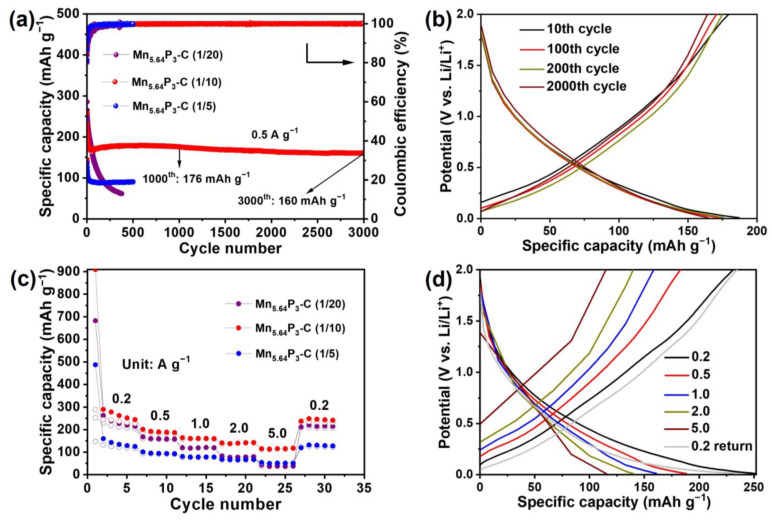
(**a**) Cycling performance at a current density of 0.5 A g^−1^ for as-synthesized Mn_5.64_P_3_ electrodes with various ratio of Mn:C, and the corresponding (**b**) galvanostatic discharge/charge voltage profiles. (**c**) Rate performance at various current densities of 0.2/0.5/1.0/2.0/5.0/0.2 A g^−1^ for as-synthesized Mn_5.64_P_3_ electrodes with various ratio of Mn:C, and the corresponding (**d**) galvanostatic discharge/charge voltage profiles.

The lithium-ion storage properties of as-synthesized Mn_5.64_P_3_ hybrid were investigated in the voltage range of 0.01–2 V vs. Li/Li^+^. As shown in Figure 4a, the cycle performances of as-synthesized Mn_5.64_P_3_ hybrid electrodes at a current density of 0.5 A g^−1^ were presented. The capacity loss can be observed in the initial several cycles in the as-synthesized Mn_5.64_P_3_ hybrid electrodes. The initial capacity loss is generally relative with the side reactions and the inevitable formation of solid electrolyte interface (SEI) films. After the initial capacity loss, the Mn_5.64_P_3_ prepared with the ratio of 1:5 of Mn:C electrode showed stable and reversible capacity of 89 mA h g^−1^ with high Coulombic efficiency (CE) of about 100%. For the Mn_5.64_P_3_ prepared with the ratio of 1:20 of Mn:C electrode, it shows higher initial capacity than that of the Mn_5.64_P_3_ prepared with the ratio of 1:5 of Mn:C electrode. However, the capacity gradually decreased and only 64 mA h g^−1^ was retained after 350 cycles with low cycle retention. Compared to these electrodes, the Mn_5.64_P_3_ prepared with the ratio of 1:10 of Mn:C electrode presents striking cycling stability at the current density of 0.5 A g^−1^. As shown in Figure 4a, the reversible capacity is as high as 176 mAh g^−1^ at a current density of 0.5 A g^−1^ after 1000 cycles and maintains 160 mAh g^−1^ after 3000 cycles, with the high-capacity retention corresponding to 91.3% of the 1000th cycle capacity. Figure 4b presents the charge–discharge voltage profiles of the Mn_5.64_P_3_ prepared with the ratio of 1:10 of Mn:C electrode at the current density of 0.5 A g^−1^. The electrode manifests a high reversible capacity of 180, 171, 175 and 164 mAh g^−1^ after 10, 100, 200, 2000 cycles at 0.5 A g^−1^, respectively, without noticeable capacity attenuation. In comparison, the charge–discharge voltage profiles of the Mn_5.64_P_3_ prepared with the ratio of 1:20 of Mn:C electrode at the current density of 0.5 A g^−1^ in Figure S6a shows dramatic capacity attenuation from the 10th cycle to 200th cycle and large potential gap. On the other hand, the discharge/charge curves of the Mn_5.64_P_3_ prepared with the ratio of 1:5 of Mn:C electrode at the current density of 0.5 A g^−1^ in Figure S6b remain stable but deliver lower capacities than those of the Mn_5.64_P_3_ prepared with the ratio of 1:10 of Mn:C electrode. The terrible cycling stability of Mn_5.64_P_3_-C (1/20) is likely caused by granule conglomeration and electrolyte consumption during repeated charge-discharge cycles. The lack of porosity in Mn_5.64_P_3_-C (1/5) and the poor mechanical property of Mn_5.64_P_3_-C (1/20) might provide insights into the short lifespan of these two electrodes. We have listed the capacity and capacitance retention of this work compared with the previously reported metal phosphates electrodes. As shown in Table S3, the obtained cycling performances in the Mn_5.64_P_3_-C electrodes are superior to most of the previously reported metal phosphates anodes.

The rate capabilities of as-synthesized Mn_5.64_P_3_ hybrid electrodes are shown in Figure 4c. At current densities of 0.2, 0.5, 1.0, 2.0, and 5.0 A g^−1^, the reversible capacities of the Mn_5.64_P_3_ prepared with the ratio of 1:10 of Mn:C electrode are about 231, 183, 159, 140, and 115 mAh g^−1^, respectively. After decreasing the current rate back to 0.2 A g^−1^, the capacity is increased to 235 mAh g^−1^, suggesting the excellent rate capability. It could be ascribable to the activation generated by discharging/charging cycles at higher rates [33]. And the discharge/charge curves of the Mn_5.64_P_3_ prepared with the ratio of 1:10 of Mn:C electrode cycling at various current densities in Figure 4d show similar processes, indicating stable storage mechanism of the Mn_5.64_P_3_ prepared with the ratio of 1:10 of Mn:C electrode at various current densities. However, the Mn_5.64_P_3_ prepared with the ratio of 1:20 of Mn:C electrode and the Mn_5.64_P_3_ prepared with the ratio of 1:5 of Mn:C electrode at various current densities present low capacities at high current densities but good capacity recovery ability at back 0.2 A g^−1^ in Figure 4c and Figure S7.

**Figure 5:**
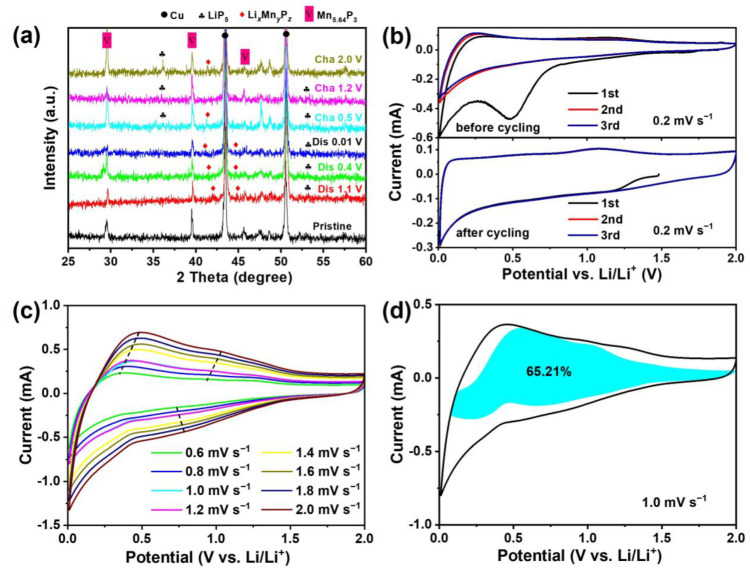
(**a**) XRD patterns of the Mn_5.64_P_3_ anodes during the initial lithiation and delithiation process at different voltages. (**b**) The cyclic voltammograms curves of Mn_5.64_P_3_-C before and after long-term cycling at the scan rate of 0.2 mV s^−1^. (**c**) CV curves of Mn_5.64_P_3_ at scan rates range from 0.6 to 2 mV s^−1^. (**d**) Pseudo-capacitance contribution at 1.0 mV s^−1^ of Mn_5.64_P_3_ anode.

In order to figure out the phase transitions during the initial lithiation and delithiation process at different voltages, ex situ XRD experiments at selected voltages were carried out. Figure 5a indicates the XRD patterns of the Mn_5.64_P_3_ prepared with the ratio of 1:10 of Mn:C electrode during the initial lithiation/delithiation process at different voltages. Before discharged, only the crystalline Mn_5.64_P_3_ phase except the copper current collector (43.3° and 50.4°) can be found. During discharging to 1.1, 0.4 and 0.01 V, the intensities of the peaks indexed to the Mn_5.64_P_3_ phase is gradually decreasing. And it can be observed that the broadening of the diffraction peaks (40–45°) are observed, and gradually shift to the smaller angles. These humps (40–45°) might be indexed to an amorphous Li–Mn–P phase, which could be denoted as Li_x_Mn_y_P_z_ [24]. Simultaneously, the another appeared hump might be indexed to a LiP_5_ phase [25]. During the subsequent charging process, the peaks indexed to the Mn_5.64_P_3_ phase are clearly observed, and gradually become stronger from 0.01 to 2.00 V. Additionally, the Li_x_Mn_y_P_z_ and LiP_5_ phase reserved during the delithiation process. The above results imply that during the initial lithiation/delithiation process the Mn_5.64_P_3_ phase was transformed to the amorphous Li_x_Mn_y_P_z_ and LiP_5_ phase to some extent, which might lead to some irreversibility capacity degradation. Generally, the generated Li_x_Mn_y_P_z_ and the LiP_5_ phases during the initial lithiation and delithiation process could act as the buffer matrix for the Mn_5.64_P_3_ active material to suppress the decrease of the lithiation and delithiation capacity during cycling. Further, Figure S8 presents the XRD patterns of the Mn_5.64_P_3_ prepared with the ratio of 1:10 of Mn:C electrode before and after cycling at 0.5 A g^−1^. It can be observed that obvious peaks indexed to the Mn_5.64_P_3_ phase and nearly no change than these peaks of the electrode before cycling, indicating robust and reversible ion storage process in Mn_5.64_P_3_ phase.

Figure 5b presents CV curves at 0.2 mV s^−1^ before and after long-term cycling to evaluate the redox activity of the electrode. The irreversible reduction peak at around 0.5 V can be observed during the first scanning process, which might be caused by the side reaction between the electrolyte and electrode. The 2nd and 3rd CV curves before cycling are not overlapped, which could be due to the gradual activation process during the initial several cycles. On the other hand, the CV curves after cycling are completely overlapped, which can indicate reversible oxidation-reduction reactions and high-capacity retention after activation process. In addition, the redox peaks at around 0.01V and 1.1 V are well-preserved before and after cycling, which could confirm the redox activity of the electrode. Figure S9 shows the electrochemical impedance spectroscopy (EIS) of Mn_5.64_P_3_ electrodes before cycling, after the 500th and 3000th cycles at 0.5 A g^−1^. The inset in Figure S9 is the equivalent circuit of the EIS fitting. Rs is the ohmic resistance of the electrode. Rsei is the resistance of lithium-ion diffusion through the SEI film. And Rct is the charge transfer resistance. CPE is the constant phase elements. W is the warburg impedance. According to the fitting equivalent circuit, the resistances of electrode after cycling significantly decrease than that of the electrode before cycling. Significantly, the Rs, Rsei and Rct resistances of electrode after 3000 cycles are 1.9 Ω, 22.2 Ω, and 75.2 Ω, respectively, which are close to the corresponding resistances of 1.5 Ω, 21.6 Ω, and 69.7 Ω after 500 cycles. Further, we have conducted the SEM results before and after the long-term cycle. As shown in Figure S10, all electrodes present flat surfaces before cycling (Figure S10a–e). However, aggregate particles, side reaction products and electrolyte consumption are observed on the Mn_5.64_P_3_-C (1/20) electrodes and the Mn_5.64_P_3_-C (1/5) electrodes after the long-term cycle (Figure S10b,f). Differently, the Mn_5.64_P_3_-C (1/10) electrode presents stable surface and contains abundant electrolyte (Figure S10d).

To furtherly study lithium storage performance of as-synthesized Mn_5.64_P_3_ electrode, a series of CV curves at various scan rates of the Mn_5.64_P_3_ prepared with the ratio of 1:10 of Mn:C electrode present in Figure 5c. The CV curves tested at different sweep rates from 0.6 to 2.0 mV s^−1^ are similar. The area of closed CV curves represents the total charge storage including the pseudo-capacitive contribution and diffusion contribution. The capacitive contribution can be calculated by the equation (*i* = k_1_*v* + k_2_*v*^1/2^) [34]. The pseudo-capacitive contribution (shaded region) of the Mn_5.64_P_3_ prepared with the ratio of 1:10 of Mn:C electrode at the scan rate of 1.0 mV s^−1^ is observed in Figure 5d. And the percentage of pseudo-capacitive contribution at 0.6, 0.8, 1.0, 1.2, 1.4, 1.6, 1.8 and 2.0 mV s^−1^ are 58.06%, 60.70%, 65.21%, 74.01%, 68.38%, 70.17%, 70.67% and 71.43%, respectively, as shown in Figure S11. The Mn_5.64_P_3_ exhibited the similar surface capacitive contributions with those of previously reported electrodes [29,35], which could be attributed to the surface spin capacitance of Mn and P in the Mn_5.64_P_3_ electrode did not significantly contribute to the total reversible capacity.

## 3. Experimental Section

### 3.1. Synthesis of the Mn_5.64_P_3_-C Hybrid

All chemical reagents (Shanghai Aladdin Biochemical Technology Co., LTD, Shanghai, China) were directly used in experiments without any further purification. First, 10 mmol melamine powder (Shanghai Aladdin Biochemical Technology Co., LTD, Shanghai, China) and 1 mmol Mn(CH_3_COO)_2_·4H_2_O powder (Shanghai Aladdin Biochemical Technology Co., LTD, Shanghai, China) were mixed by grinding in the crucible. Then, the mixture was stirred with 4 mL of phytic acid (Shanghai Aladdin Biochemical Technology Co., LTD, Shanghai, China) for 10 min and dried in an oven at 90 °C for 30 min. The negative phosphate groups in phytic acid (C_6_H_18_O_24_P_6_) with strong chelating ability could easily bond with positive manganese ions. In addition, the hydrogen bonding between amino group of melamine and phosphate groups of phytic acid [30]. For the preparation of Mn_5.64_P_3_-C, the precursors were heat treated at 900 °C for 3 h in argon atmosphere with the heating rate of 5 °C min^−1^. The attained Mn_5.64_P_3_-C hybrid is denoted as Mn_5.64_P_3_-C (1/10). The other contrastive Mn_5.64_P_3_-C hybrids with different additive amounts of carbon sources were also synthesized by a similar procedure except for using 5 mmol melamine (denoted as Mn_5.64_P_3_-C (1/5)) and 20 mmol melamine (denoted as Mn_5.64_P_3_-C (1/20)).

### 3.2. Materials Characterization

The crystalline structures of as-synthesized Mn_5.64_P_3_-C hybrid materials were investigated by XRD (Bruker D8 Advance, Cu Kα radiation, Bruker AXS GmbH, Karlsruhe, Germany). In addition, the microstructure of as-synthesized Mn_5.64_P_3_-C hybrid materials were characterized by the field-emission scanning electron microscope (FESEM, GeminiSEM 500, Carl Zeiss AG, Oberkochen, Germany) and the field emission transmission electron microscope (TEM, JEOL ARM-200F, Hitachinaka, Naka, Japan). XPS measurements were conducted on an ESCALAB 250Xi (Thermo-VG Scientific, Waltham, MA, USA) to figure out the chemical state of the surface elements in Mn_5.64_P_3_-C hybrid materials. The specific surface area of sample was determined by using the Brunauer–Emmett–Teller (BET) equation based on the N_2_ adsorption–desorption isotherms (Mike ASAP-2460, Micromeritics Instrument Corporation, Norcross, United States). The Raman spectra were recorded in the JY LABRAM-HR confocal laser micro-Raman spectrometer (HORIBA Scientific, Palaiseau, France) to investigate the order of carbon materials in the prepared Mn_5.64_P_3_-C hybrid materials.

### 3.3. Electrochemical Measurements

The electrochemical performances of as-synthesized Mn_5.64_P_3_-C hybrid materials in LIBs were evaluated in half-cell configuration. The slurries were prepared by mixing the as-synthesized Mn_5.64_P_3_-C hybrid materials (70 wt%), poly(vinyl difluoride) (10 wt%), and acetylene black (20 wt%), and then coating on copper foil. The mass loading of the prepared electrode material with a diameter of 12 mm was ≈1.0 mg cm^−1^. The two-electrode 2032-coin cells were assembled in Ar-filled glovebox with lithium foil as the counter/reference electrode and Celgard 2400 membrane as the separator. The electrolyte was LiPF_6_ (1 mol) in a mixture (*v/v/v* = 1:1:1) of ethylene carbonate, dimethyl carbonate, and diethyl carbonate. The charge–discharge tests were performed on Neware battery test systems (BTS-5V20mA, Shenzhen, China) between 0.01 and 2 V at various current densities. Electrochemical impedance spectroscopy (EIS) analysis was conducted at the frequency range of 100 kHz to 0.01 Hz. CV tests with different scan rates were measured using the CHI 660 electrochemical workstation (Chenhua, Shanghai, China).

## 4. Conclusions

In summary, the Mn_5.64_P_3_ with hexagonal crystal structure embedded into N/P co-doped porous carbon shells are successfully synthesized through a self-template and recrystallization–self-assembly strategy. The submicron Mn_5.64_P_3_ particles can uniformly dispersed in porous carbon shells with the ratio of 1:10 of Mn:C. And the N/P co-doped porous carbon shells can provide high specific capacity for electrolyte infiltration. After the initial irreversible capacity loss because of side reactions, the Mn_5.64_P_3_-C anode delivers superior cycling performance with 160 mAh g^−1^ at 0.5 A g^−1^ after 3000 cycles with the 91.3% of the capacity in 1000th cycle, and present high-rate capacity of 115 mAh g^−1^ at high current density of 5.0 A g^−1^. The synergistic effect of robust Mn_5.64_P_3_ with hexagonal crystal structure and porous carbon shells are believed to be responsible for the satisfactory cycling performance and rate capability. The Mn_5.64_P_3_ with hexagonal crystal structure and N/P co-doped doped carbon shells are believed to be responsible for the superior cycling performance. The design of the Mn_5.64_P_3_-C hybrid could promote the further optimization and development of manganese phosphide for high-performance LIBs and other electrochemical applications.

## Data Availability

The data presented in this study are available on request from the corresponding author.

## Supplementary Materials

The following supporting information can be downloaded at: https://www.mdpi.com/article/10.3390/molecules30061346/s1, Figure S1: the crystal structure of MnP; Figure S2: XRD Rietveld refinement results of as-synthesized Mn_5.64_P_3_-C (1/20); Figure S3: XRD Rietveld refinement results of as-synthesized Mn_5.64_P_3_-C (1/5); Figure S4: the SEM images of the as-synthesized Mn_5.64_P_3_ with the ratio of 1:5 of Mn:C; Figure S5: the SEM images of the as-synthesized Mn_5.64_P_3_ with the ratio of 1:20 of Mn:C; Figure S6: (a) Galvanostatic discharge/charge voltage profiles of the as-synthesized Mn_5.64_P_3_ with the ratio of 1:20 of Mn:C at 0.5 A g^−1^, (b) galvanostatic discharge/charge voltage profiles of the as-synthesized Mn_5.64_P_3_ with the ratio of 1:5 of Mn:C at 0.5 A g^−1^; Figure S7: (a) Galvanostatic discharge/charge voltage profiles of the as-synthesized Mn_5.64_P_3_ with the ratio of 1:20 of Mn:C at various current densities of 0.2/0.5/1.0/2.0/5.0/returned 0.2 A g^−1^, (b) galvanostatic discharge/charge voltage profiles of the as-synthesized Mn5.64P3 with the ratio of 1:5 of Mn:C at various current densities of 0.2/0.5/1.0/2.0/5.0/returned 0.2 A g^−1^; Figure S8: XRD patterns of the Mn_5.64_P_3_ anodes before cycle and after the 3000^th^ cycle; Figure S9: electrochemical impedance spectroscopy (EIS) data for Mn_5.64_P_3_ electrodes before cycle, after the 500th and 3000th cycles at a current density of 0.5 A g^−1^; Figure S10: SEM images before and after the long-term cycle of as-synthesized Mn_5.64_P_3_-C (1/20) electrodes (a, b), Mn_5.64_P_3_-C (1/10) electrodes (c, d) and Mn_5.64_P_3_-C (1/5) electrodes (e, f), respectively; Figure S11: (a) Pseudo-capacitance contribution at 0.6 mV s^−1^ of Mn_5.64_P_3_, (b) pseudo-capacitance contribution at 0.8 mV s^−1^ of Mn_5.64_P_3_, (c) pseudo-capacitance contribution at 1.2 mV s^−1^ of Mn_5.64_P_3_, (d) pseudo-capacitance contribution at 1.4 mV s^−1^ of Mn_5.64_P_3_, (e) pseudo-capacitance contribution at 1.6 mV s^−1^ of Mn_5.64_P_3_, (f) pseudo-capacitance contribution at 1.8 mV s^−1^ of Mn_5.64_P_3_, (g) pseudo-capacitance contribution at 2.0 mV s^−1^ of Mn_5.64_P_3_, (h) pseudo-capacitance ratio at different sweep speeds of Mn_5.64_P_3_. Table S1: the refined lattice parameters for as-synthesized Mn_5.64_P_3_ with various ratio of Mn:C; Table S2: atomic position parameters of as-synthesized Mn_5.64_P_3_ with various ratio of Mn:C; Table S3: lithium storage performance comparison of Mn_5.64_P_3_-C electrode with the previously reported metal phosphates electrodes [24,25,26,28,29,36].

## References

[1] Degen F., Winter M., Bendig D., Tübke J. (2023). Energy consumption of current and future production of lithium-ion and post lithium-ion battery cells. Nat. Energy.

[2] Li J.L., Fleetwood J., Hawley W.B., Kays W. (2022). From Materials to Cell: State-of-the-Art and Prospective Technologies for Lithium-Ion Battery Electrode Processing. Chem. Rev..

[3] Gao Y.-M., Liu Y., Feng K.-J., Ma J.-Q., Miao Y.-J., Xu B.-R., Pan K.-M., Akiyoshi O., Wang G.-X., Zhang K.-K. (2024). Emerging WS_2_/WSe_2_@graphene nanocomposites: Synthesis and electrochemical energy storage applications. Rare Met..

[4] Fang S., Bresser D., Passerini S. (2020). Transition Metal Oxide Anodes for Electrochemical Energy Storage in Lithium- and Sodium-Ion Batteries. Adv. Energy Mater..

[5] Liu Y., Wang Y., Wang F., Lei Z., Zhang W., Pan K., Liu J., Chen M., Wang G., Ren F. (2019). Facile Synthesis of Antimony Tungstate Nanosheets as Anodes for Lithium-Ion Batteries. Nanomaterials.

[6] Liang S.Z., Cheng Y.J., Zhu J., Xia Y.G., Müller-Buschbaum P. (2020). A Chronicle Review of Nonsilicon (Sn, Sb, Ge)-Based Lithium/Sodium-Ion Battery Alloying Anodes. Small Methods.

[7] Peng M.Q., Shin K., Jiang L.X., Jin Y., Zeng K., Zhou X.L., Tang Y.B. (2022). Alloy-Type Anodes for High-Performance Rechargeable Batteries. Angew. Chem.-Int. Ed..

[8] Qian H., Liu Y., Chen H., Feng K., Jia K., Pan K., Wang G., Huang T., Pang X., Zhang Q. (2023). Emerging bismuth-based materials: From fundamentals to electrochemical energy storage applications. Energy Storage Mater..

[9] Hu Y.W., Yang H., Balogun M.S., Chen J. (2023). Understanding the lithium storage mechanism of free-standing Fe_2_N nanoparticles. Inorg. Chem. Commun..

[10] Zhang D., Zhang C.Y., Xu H.S., Huo Z., Shi X.Y., Liu X.D., Liu G.Y., Yu C. (2024). Facilely Fabricating F-Doped Fe_3_N Nanoellipsoids Grown on 3D N-Doped Porous Carbon Framework as a Preeminent Negative Material. Molecules.

[11] Chen Y.F., Wang J.Z., Hong Y.R., Yang Y.S., Tan L.L., Li N., Ma C., Wang J.W., Fan X.L., Zhu Y.J. (2023). Uncovering the untapped potential of copper(I) sulphide toward lithium-ion storage under ultra-low temperatures. J. Mater. Chem. A.

[12] Lenus S., Yaoda L., Thakur P., Lal A., Samantaray S.S., Dai Z.F., Narayanan T.N. (2024). Hydrated LiOH modified Ni_0.1_Fe_0.9_PS_3_ anodes towards safer high-performance lithium-ion batteries. Electrochim. Acta.

[13] Chen F.Z., Xu J., Wang S.Y., Lv Y.H., Li Y., Chen X., Xia A.L., Li Y.T., Wu J.X., Ma L.B. (2022). Phosphorus/Phosphide-Based Materials for Alkali Metal-Ion Batteries. Adv. Sci..

[14] Lan X.X., Li Z., Zeng Y., Han C.P., Peng J., Cheng H.M. (2024). Phosphorus-based anodes for fast-charging alkali metal ion batteries. Ecomat.

[15] He R.X., Wang X.X., Li J.H., Yao F., Wang H.R., Nie P., Chang L.M. (2024). Three-dimensional porous structure CoP/N-doped carbon nanospheres as anode for enhanced lithium storage performance. J. Energy Storage.

[16] Mei P., Pramanik M., Young C., Huang Z.G., Hossain M.S.A., Sugahara Y., Yamauchi Y. (2017). Synthesis of mesostructured manganese phosphonate and its promising energy storage application. J. Mater. Chem. A.

[17] Bi W.C., Zhang L.F., Chen J., Tian R.X., Huang H., Yao M. (2022). Lithiation Mechanism and Performance of Monoclinic ZnP_2_ Anode Materials. Acta Chim. Sin..

[18] He R.X., Wang X.X., Li J.H., Chang L.M., Wang H.R., Nie P. (2024). Engineering ultra-small tin phosphide encapsulated in 3D phosphorous-doped porous carbon nanosheets as high-performance anodes for lithium-ion batteries. Appl. Surf. Sci..

[19] Zhang X., Lv B., Peng Y.H., Li Q., Chen M., Liu X.G., Wei Y.Y., Zuo X.J. (2025). Elastic restraint induced by mesoporous carbon tubes of ultrafine Ni_2_P nanoparticles for enhanced potassium and lithium storage. Appl. Surf. Sci..

[20] Ou H., Li P., Jiang C.Y., Liu Y.Q., Luo Y.H., Xing Z.Y., Zeb A., Wu Y.B., Lin X.M. (2025). Synergistic enhancement of Ni_2_P anode for high lithium/sodium storage by N, P, S triply-doping and soft template-assisted strategy. J. Colloid Interface Sci..

[21] Zhong J.J., Li J.L. (2024). Copper Phosphide Nanostructures Covalently Modified Ti_3_C_2_T_x_ for Fast Lithium-Ion Storage by Enhanced Kinetics and Pesudocapacitance. Small.

[22] Shen Y.H., Jiang Y.L., Yang Z.Z., Dong J., Yang W., An Q.Y., Mai L.Q. (2022). Electronic Structure Modulation in MoO_2_/MoP Heterostructure to Induce Fast Electronic/Ionic Diffusion Kinetics for Lithium Storage. Adv. Sci..

[23] Liu Y.D., Gao S.Y., Zhang D.Y., Niu X.H., Li H.X., Wang K.J. (2024). In-suit synthesis of FeP/C/CNT composites with excellent performance in supercapacitors and lithium-ion batteries. Chemistryselect.

[24] Li L., Peng Y., Yang H. (2013). Phase structure changes of MnP anode material during electrochemical lithiation and delithiation process. Electrochim. Acta.

[25] Sim S., Cho J. (2012). Li Reaction Mechanism of MnP Nanoparticles. J. Electrochem. Soc..

[26] Mei P., Lee J., Pramanik M., Alshehri A., Kim J., Henzie J., Kim J.H., Yamauchi Y. (2018). Mesoporous Manganese Phosphonate Nanorods as a Prospective Anode for Lithium-Ion Batteries. ACS Appl. Mater. Interfaces.

[27] Gillot F., Monconduit L., Morcrette M., Doublet M.L., Dupont L., Tarascon J.M. (2005). On the Reactivity of Li_8-y_Mn_y_P_4_ toward Lithium. Chem. Mater..

[28] Kim K.-H., Kim W.-S., Hong S.-H. (2019). Solid solution phosphide (Mn_1−x_F_ex_P) as a tunable conversion/alloying hybrid anode for lithium-ion batteries. Nanoscale.

[29] Kim K.-H., Oh J., Jung C.-H., Kim M., Gallant B.M., Hong S.-H. (2021). A Novel Solid Solution Mn_1-x_V_x_P Anode with Tunable Alloying/Insertion Hybrid Electrochemical Reaction for High Performance Lithium Ion Batteries. Energy Storage Mater..

[30] Bai J., Xi B., Mao H., Lin Y., Ma X., Feng J., Xiong S. (2018). One-Step Construction of N,P-Codoped Porous Carbon Sheets/CoP Hybrids with Enhanced Lithium and Potassium Storage. Adv. Mater..

[31] Hong X., Deng C., Wang G., Wang X., Dong W. (2023). Composition and morphology transition of NF/MnP/NiCoP composite electrode induced by charge/discharge activation. Chem. Eng. J..

[32] Zhang X., Yang F., Sun S., Wei K., Liu H., Li G., Sun Y., Li X., Qian J., Du S. (2024). Boosting oxygen reduction via MnP nanoparticles encapsulated by N, P-doped carbon to Mn single atoms sites for Zn-air batteries. J. Colloid Interface Sci..

[33] Forney M.W., Ganter M.J., Staub J.W., Ridgley R.D., Landi B.J. (2013). Prelithiation of Silicon–Carbon Nanotube Anodes for Lithium Ion Batteries by Stabilized Lithium Metal Powder (SLMP). Nano Lett..

[34] Liu S., Zheng W., Xie W., Cui H., Li Y., Zhang C., Ji Z., Liu F., Chen R., Sun H. (2022). Synthesis of three-dimensional honeycomb-like Fe_3_N@NC composites with enhanced lithium storage properties. Carbon.

[35] Haghighat-Shishavan S., Nazarian-Samani M., Nazarian-Samani M., Roh H.-K., Chung K.-Y., Cho B.-W., Kashani-Bozorg S.F., Kim K.-B. (2018). Strong, persistent superficial oxidation-assisted chemical bonding of black phosphorus with multiwall carbon nanotubes for high-capacity ultradurable storage of lithium and sodium. J. Mater. Chem. A.

[36] Chakraborty D., Dam T., Modak A., Pant K.K., Chandra B.K., Majee A., Ghosh A., Bhaumik A. (2021). A novel crystalline nanoporous iron phosphonate based metal–organic framework as an efficient anode material for lithium ion batteries. New J. Chem..

