# Supplementary material for: A Novel N/P-Doped Carbon Shells/Mn5.64P3 with Hexagonal Crystal Structure Hybrid as a Prospective Anode for Lithium-Ion Batteries
Source: Molecules. 2025 Mar 17;30(6):1346. doi: 10.3390/molecules30061346 (PMC11944552; doi:10.3390/molecules30061346)
Supplement: Supplementary file 1 [file molecules-30-01346-s001.zip › molecules-3504032-supplementary.pdf]

Supporting Information

A Novel N/P-Doped Carbon Shells/$Mn_{5.64}P_3$ with Hexagonal Crystal Structure Hybrid as a Prospective Anode for Lithium-Ion Batteries

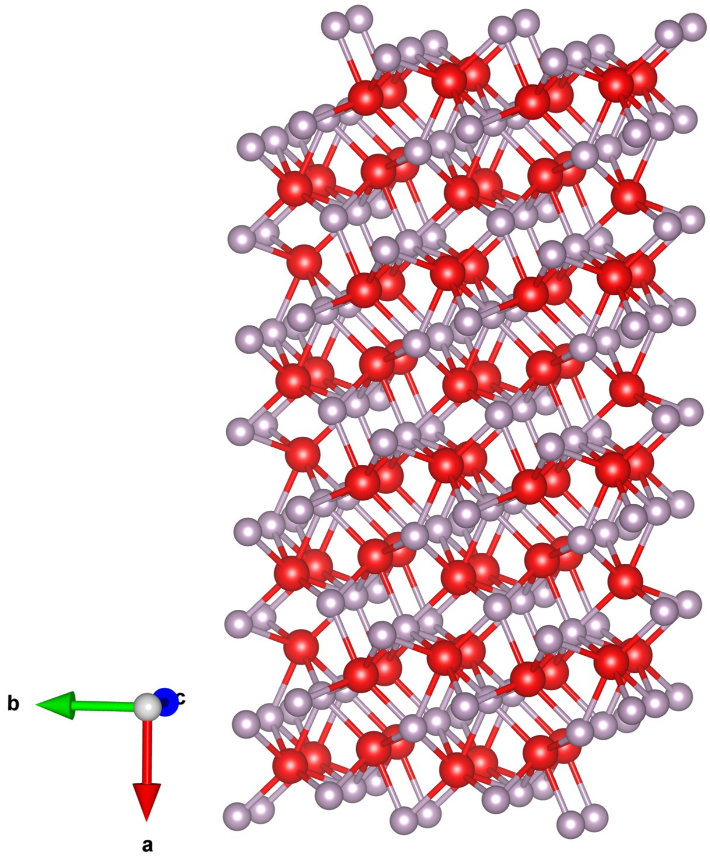

Figure S1. The crystal structure of MnP.

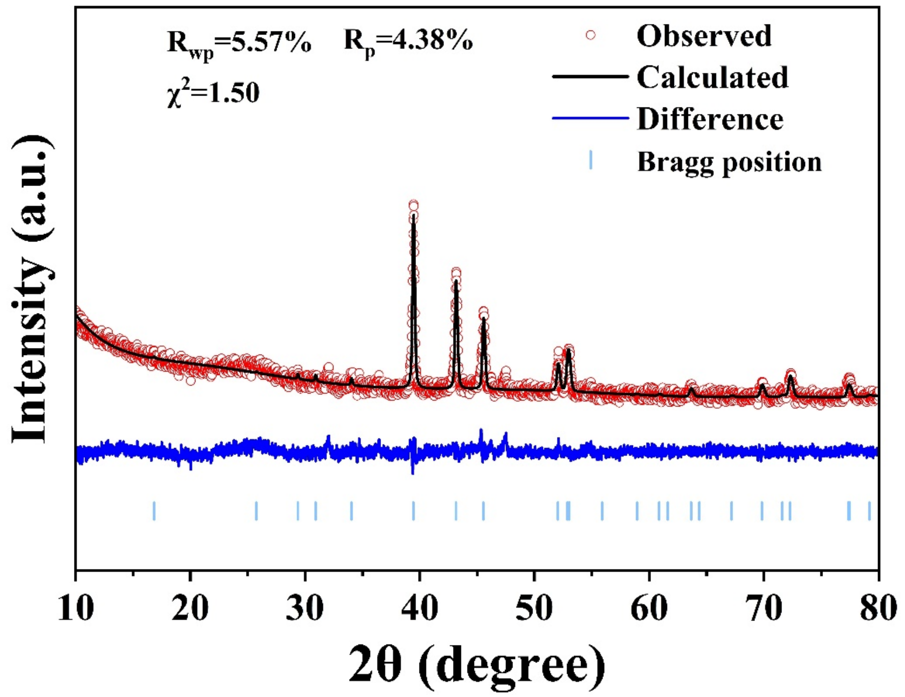

Figure S2. XRD Rietveld refinement results of as-synthesized $Mn_{5.64}P_3$-C (1/20).

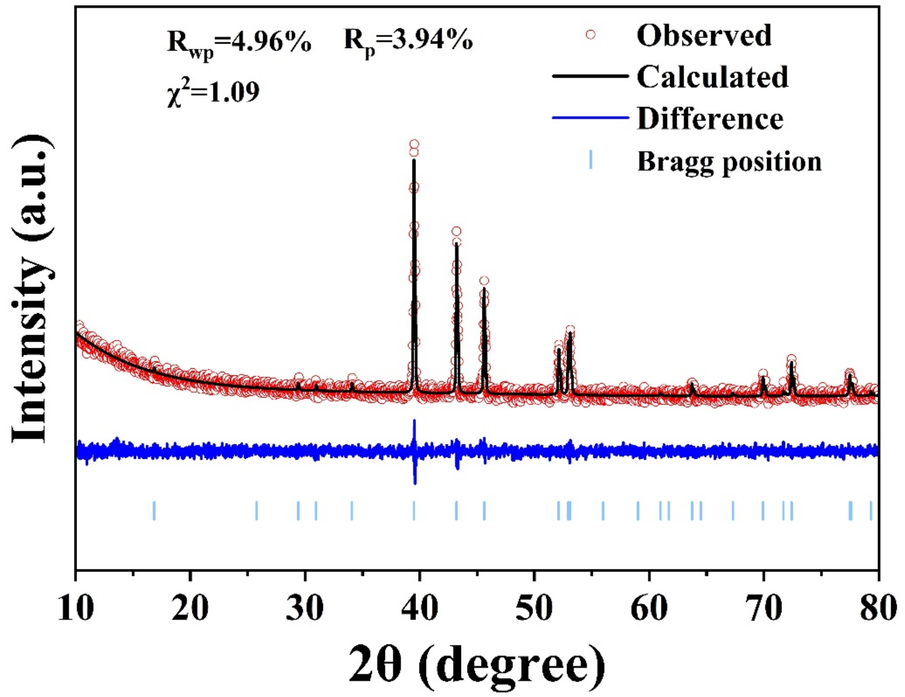

Figure S3. XRD Rietveld refinement results of as-synthesized $Mn_{5.64}P_3$-C (1/5).

Table S1. The refined lattice parameters for as-synthesized $Mn_{5.64}P_3$ with various ratio of Mn: C.

Material	*a* (Å)	*b* (Å)	*c* (Å)	*V* (Å^3)
$Mn_{5.64}P_3$-C (1/20)	6.0857(7)	6.0857	3.46225(28)	111.047(13)
$Mn_{5.64}P_3$-C (1/10)	6.0842(10)	6.0842	3.4595(4)	110.906(30)
$Mn_{5.64}P_3$-C (1/5)	6.07767(31)	6.07767	3.45714(12)	110.591(6)

Table S2. Atomic position parameters of as-synthesized $Mn_{5.64}P_3$ with various ratio of Mn: C.

Material	Atom	*x*	*y*	*z*	Occupancy
$Mn_{5.64}P_3$-C (1/20)	Mn1	0.25194	0.00000	0.00000	0.8800
	Mn2	0.59109	0.00000	0.50000	1. 0000
	P1	0.00000	0.00000	0.50000	1. 0000
	P2	0.33330	0.66670	0.00000	1. 0000
$Mn_{5.64}P_3$-C (1/10)	Mn1	0.25102	0.00000	0.00000	0.8800
	Mn2	0.59150	0.00000	0.50000	1. 0000
	P1	0.00000	0.00000	0.50000	1. 0000
	P2	0.33330	0.66670	0.00000	1.0000
$Mn_{5.64}P_3$-C (1/5)	Mn1	0.25233	0.00000	0.00000	0.8800
	Mn2	0.59486	0.00000	0.50000	1. 0000
	P1	0.00000	0.00000	0.50000	1. 0000
	P2	0.33330	0.66670	0.00000	1. 0000

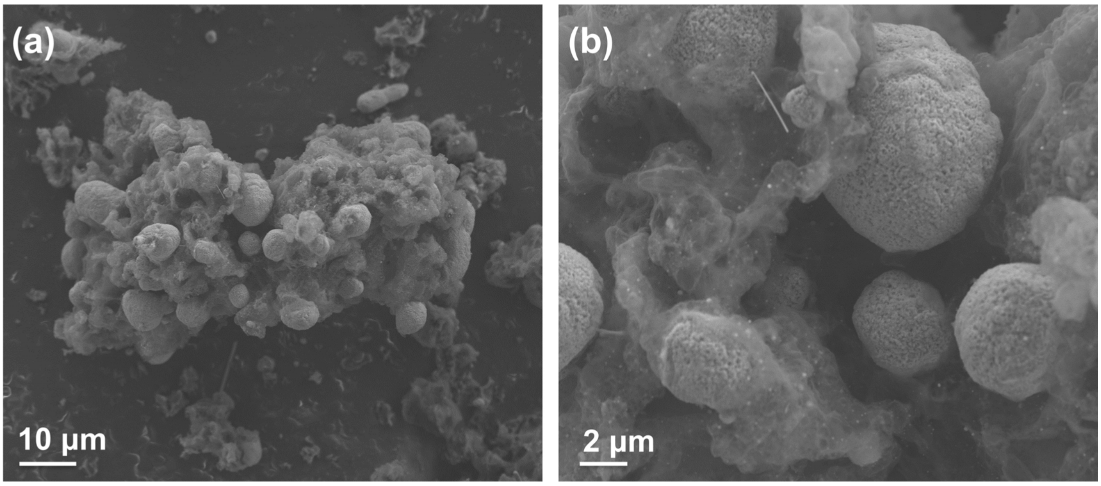

Figure S4. The SEM images of the as-synthesized $Mn_{5.64}P_3$ with the ratio of 1:5 of Mn: C.

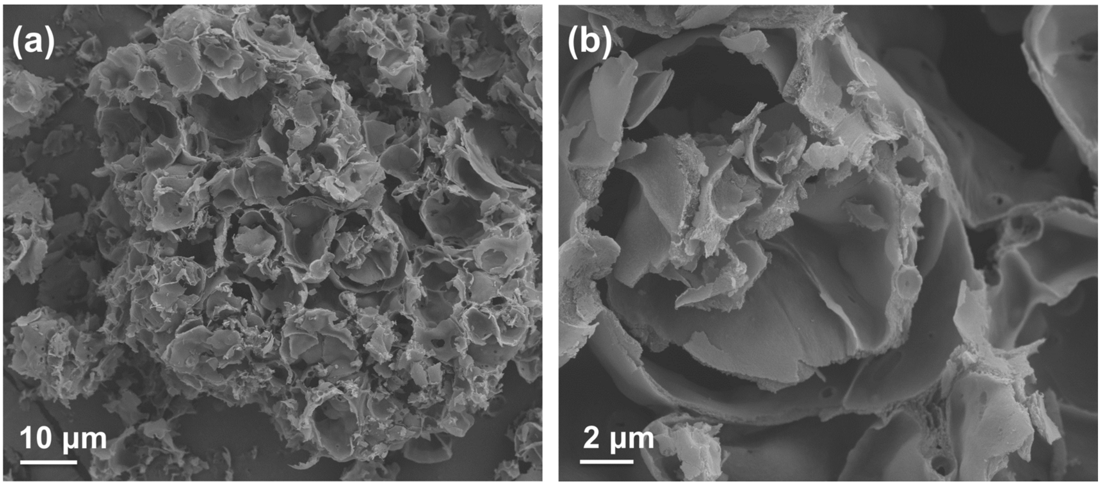

Figure S5. The SEM images of the as-synthesized $Mn_{5.64}P_3$ with the ratio of 1:20 of Mn: C.

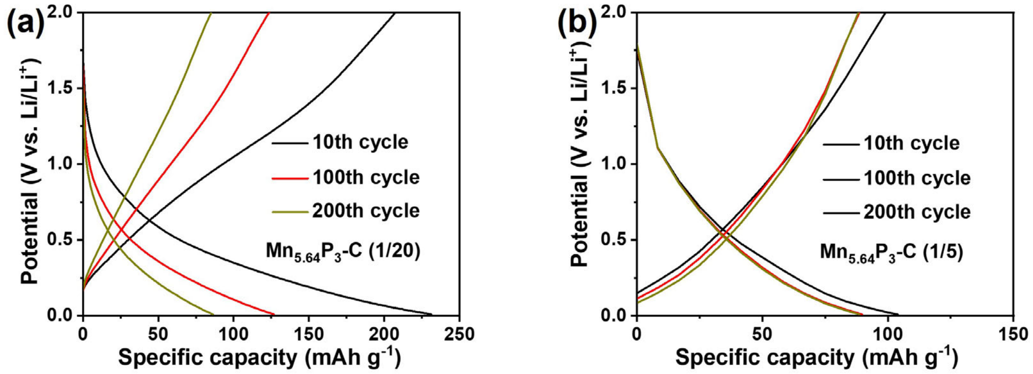

Figure S6. (a) Galvanostatic discharge/charge voltage profiles of the as-synthesized $Mn_{5.64}P_3$ with the ratio of 1:20 of Mn: C at 0.5 A g^{-1}; (b) Galvanostatic discharge/charge voltage profiles of the as-synthesized $Mn_{5.64}P_3$ with the ratio of 1:5 of Mn: C at 0.5 A g^{-1}.

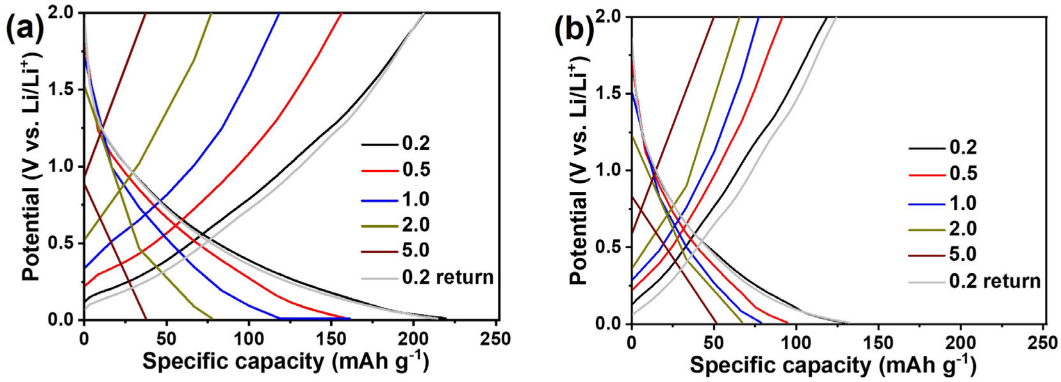

Figure S7. (a) Galvanostatic discharge/charge voltage profiles of the as-synthesized $Mn_{5.64}P_3$ with the ratio of 1:20 of Mn: C at various current densities of 0.2/0.5/1.0/2.0/5.0/returned 0.2 A g^{-1}; (b) Galvanostatic discharge/charge voltage profiles of the as-synthesized $Mn_{5.64}P_3$ with the ratio of 1:5 of Mn: C at various current densities of 0.2/0.5/1.0/2.0/5.0/returned 0.2 A g^{-1}.

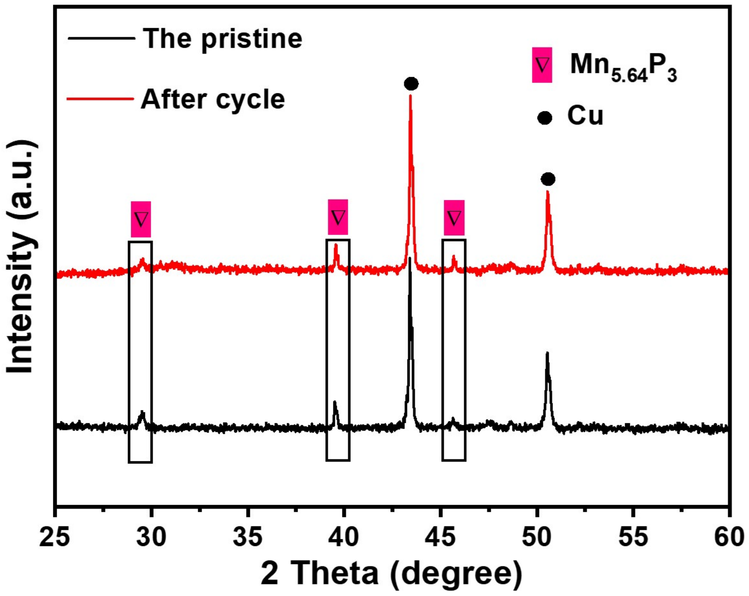

Figure S8. XRD patterns of the $Mn_{5.64}P_3$ anodes before cycle and after the 3000th cycle.

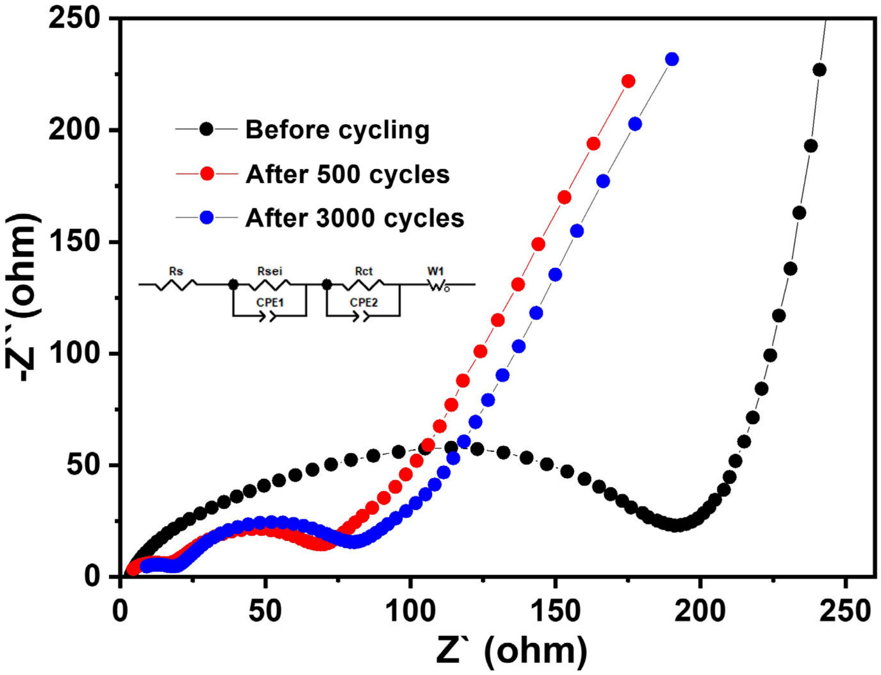

Figure S9. (a) Electrochemical impedance spectroscopy (EIS) data for $Mn_{5.64}P_3$ electrodes before cycle, after the 500th and 3000th cycles at a current density of 0.5 A g^{-1}.

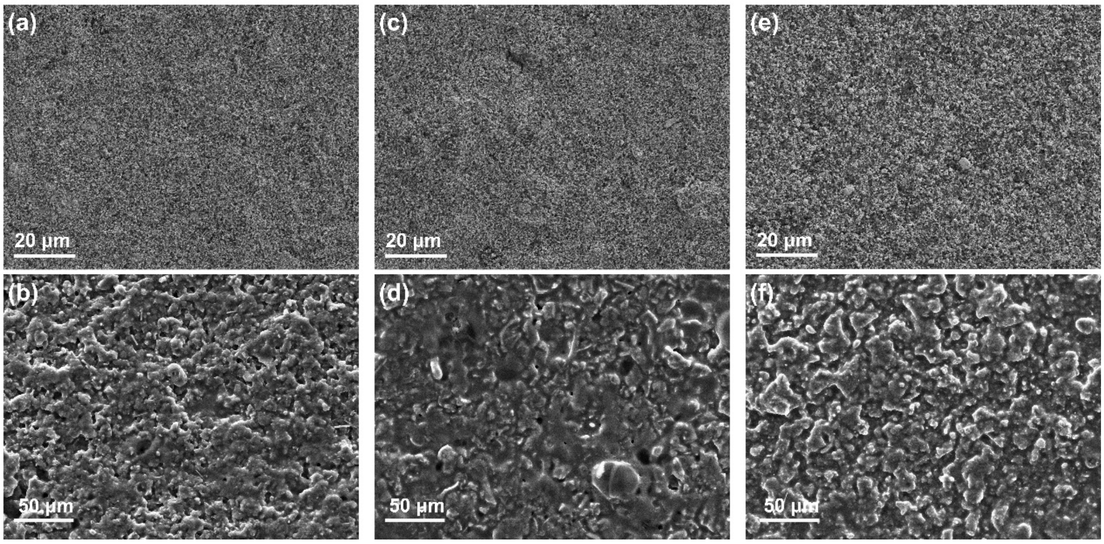

Figure S10. SEM images before and after the long-term cycle of as-synthesized $Mn_{5.64}P_3$-C (1/20) electrodes (a, b), $Mn_{5.64}P_3$-C (1/10) electrodes (c, d) and $Mn_{5.64}P_3$-C (1/5) electrodes (e, f), respectively.

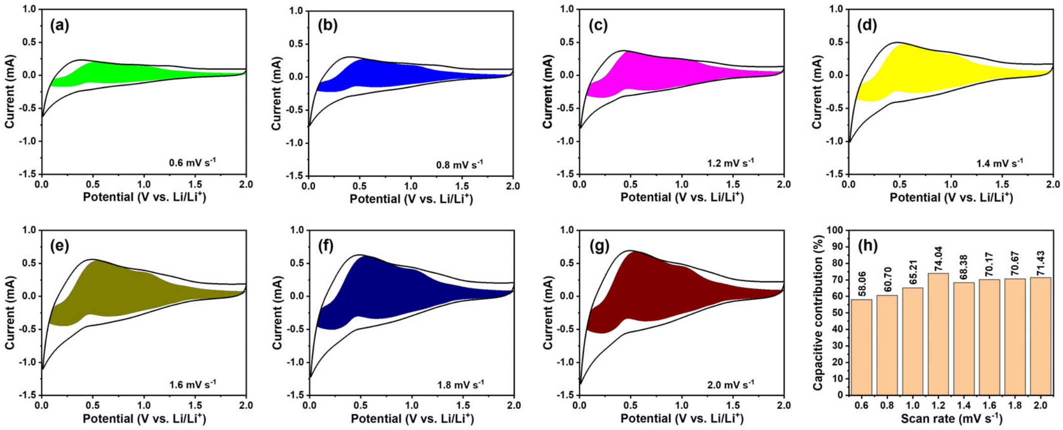

Figure S11. (a) Pseudo-capacitance contribution at 0.6 mV s^{-1} of $Mn_{5.64}P_3$; (b) Pseudo-capacitance contribution at 0.8 mV s^{-1} of $Mn_{5.64}P_3$; (c) Pseudo-capacitance contribution at 1.2 mV s^{-1} of $Mn_{5.64}P_3$; (d) Pseudo-capacitance contribution at 1.4 mV s^{-1} of $Mn_{5.64}P_3$; (e) Pseudo-capacitance contribution at 1.6 mV s^{-1} of $Mn_{5.64}P_3$; (f) Pseudo-capacitance contribution at 1.8 mV s^{-1} of $Mn_{5.64}P_3$; (g) Pseudo-capacitance contribution at 2.0 mV s^{-1} of $Mn_{5.64}P_3$. (h) Pseudo-capacitance ratio at different sweep speeds of $Mn_{5.64}P_3$.

Table S3. Lithium storage performance comparison of $Mn_{5.64}P_3$-C electrode with the previously reported metal phosphates electrodes.

**Materials**	**Current density (mA g^{-1})**	**Capacity (mAh g^{-1})**	**Cycle number (cycle retention)**	**References**
**MnP nanorod**	144	253	400 (60.2%)	[1]
**MnP powder**	50	287	50 (33%)	[2]
**MnP nanoparticle**	120	289	10 (80%)	[3]
**Nanoporous iron phosphonate**	50	275	50 (~70%)	[4]
**$Mn_{0.25}Fe_{0.75}P$**	1000	464	60 (97%)	[5]

**nanoparticle**				
**$Mn_{0.75}V_{0.25}P$ nanoparticle**	1000	321	5000 (81%)	[6]
**$Mn_{5.64}P_3$-C**	500	160	3000 (~93.6%)	This work

References:

[1] Mei, P.; Lee, J.; Pramanik, M.; Alshehri, A.; Kim, J.; Henzie, J.; Kim, J. H.; Yamauchi, Y., Mesoporous Manganese Phosphonate Nanorods as a Prospective Anode for Lithium-Ion Batteries. *ACS Applied Materials & Interfaces* **2018,** 10, (23), 19739-19745.

[2] Li, L.; Peng, Y.; Yang, H., Phase structure changes of MnP anode material during electrochemical lithiation and delithiation process. *Electrochimica Acta* **2013,** 95, 230-236.

[3] Sim, S.; Cho, J., Li Reaction Mechanism of MnP Nanoparticles. *Journal of The Electrochemical Society* **2012,** 159, (5), A669-A672.

[4] Chakraborty, D.; Dam, T.; Modak, A.; Pant, K. K.; Chandra, B. K.; Majee, A.; Ghosh, A.; Bhaumik, A., A novel crystalline nanoporous iron phosphonate based metal–organic framework as an efficient anode material for lithium ion batteries. *New Journal of Chemistry* **2021**, 45, (34), 15458-15468.

[5] Kim, K.-H.; Kim, W.-S.; Hong, S.-H., Solid solution phosphide ($Mn_{1-x}Fe_xP$) as a tunable conversion/alloying hybrid anode for lithium-ion batteries. *Nanoscale* **2019,** 11, (28), 13494-13501.

[6] Kim, K.-H.; Oh, J.; Jung, C.-H.; Kim, M.; Gallant, B. M.; Hong, S.-H., A Novel Solid Solution $Mn_{1-x}V_xP$ Anode with Tunable Alloying/Insertion Hybrid Electrochemical Reaction for High Performance Lithium Ion Batteries. *Energy Storage Materials* **2021,** 41, 310-320.
